# The expression of establishment of cohesion 1 homolog 2 (ESCO2) in tumor cells and its research progress as a therapeutic target

**DOI:** 10.1186/s40001-025-03164-4

**Published:** 2025-10-06

**Authors:** Chunmei Lu, Hanchi Wu, Huiyu Wang

**Affiliations:** 1 The Affiliated Wuxi People′s Hospital of Nanjing Medical University, Wuxi People′s Hospital, Wuxi Medical Center, Nanjing Medical University, Wuxi, China; 2 Department of Oncology, The Affiliated Wuxi People′s Hospital of Nanjing Medical University, Wuxi People′s Hospital, Wuxi Medical Center, Nanjing Medical University, 299 Qingyang Road, Wuxi, 214023 China

**Keywords:** ESCO2, Malignant tumors, Treatment

## Abstract

The ‌initiation and progression‌ of tumors are closely linked to aberrant regulation of multiple genes. Among these genes, sister chromatid cohesion acetyltransferase 2 (ESCO2) has emerged as an important target in cancer research. ESCO2 exhibits complex and heterogeneous expression patterns across various types of malignancies and tumor progression stages. Extensive studies have shown that ESCO2 primarily functions in chromosomal cohesion, significantly contributing to genomic stability maintenance. Dysregulated expression of ESCO2 in tumor cells is often correlated with malignant behaviors. Given its essential role in tumorigenesis, ESCO2 has increasingly been explored as a promising therapeutic target in recent years. This review article summarizes recent research progress regarding ESCO2 in malignant tumors, aiming to identify a novel target for their treatment.

**Supplementary Information:**

The online version contains supplementary material available at 10.1186/s40001-025-03164-4.

## Introduction

Malignant tumors are characterized by uncontrolled proliferation and the potential for metastasis, resulting from multiple etiological factors and systemic abnormalities [1]. According to recent statistics, the global annual incidence of new cancer cases has reached approximately 20 million, with cancer-related deaths approaching 9.7 million [2]. Consequently, malignant tumors represent a leading cause of Human mortality, with both morbidity and mortality rates steadily increasing. In 2022, approximately 4.8 million new cancer cases and 2.6 million cancer-related deaths occurred in China, accounting for 25.1% and 30.2% of global cases, respectively [3–5]. It is estimated that the global cancer burden will reach 28.4 million cases by 2040 [6]. Monitoring data on causes of death in China indicate that malignant tumors represent one of the main diseases threatening the health of residents, accounting for nearly one-quarter of all-cause deaths [7]. Currently, malignant tumor treatment modalities primarily include surgical resection, radiotherapy, chemotherapy, immunotherapy, and targeted therapy. However, therapeutic outcomes remain unsatisfactory. Thus, identifying novel treatment strategies is urgently needed. Recently, research on cancer-related genes has emerged as a focal area [8]. Studies have demonstrated that ESCO2 is associated with malignant tumors. Regulation of ESCO2 expression can inhibit tumor cell proliferation and progression, thereby suppressing tumor growth. This review summarizes the expression profiles of ESCO2 in diverse malignancies and the effects of regulating its expression on tumor biological functions. ESCO2 represents a promising novel therapeutic target, holding significant potential for advancing cancer therapy.

## Sister chromatid cohesion poly (**n**-acetyltransferase 2)

**Fig. 1:**
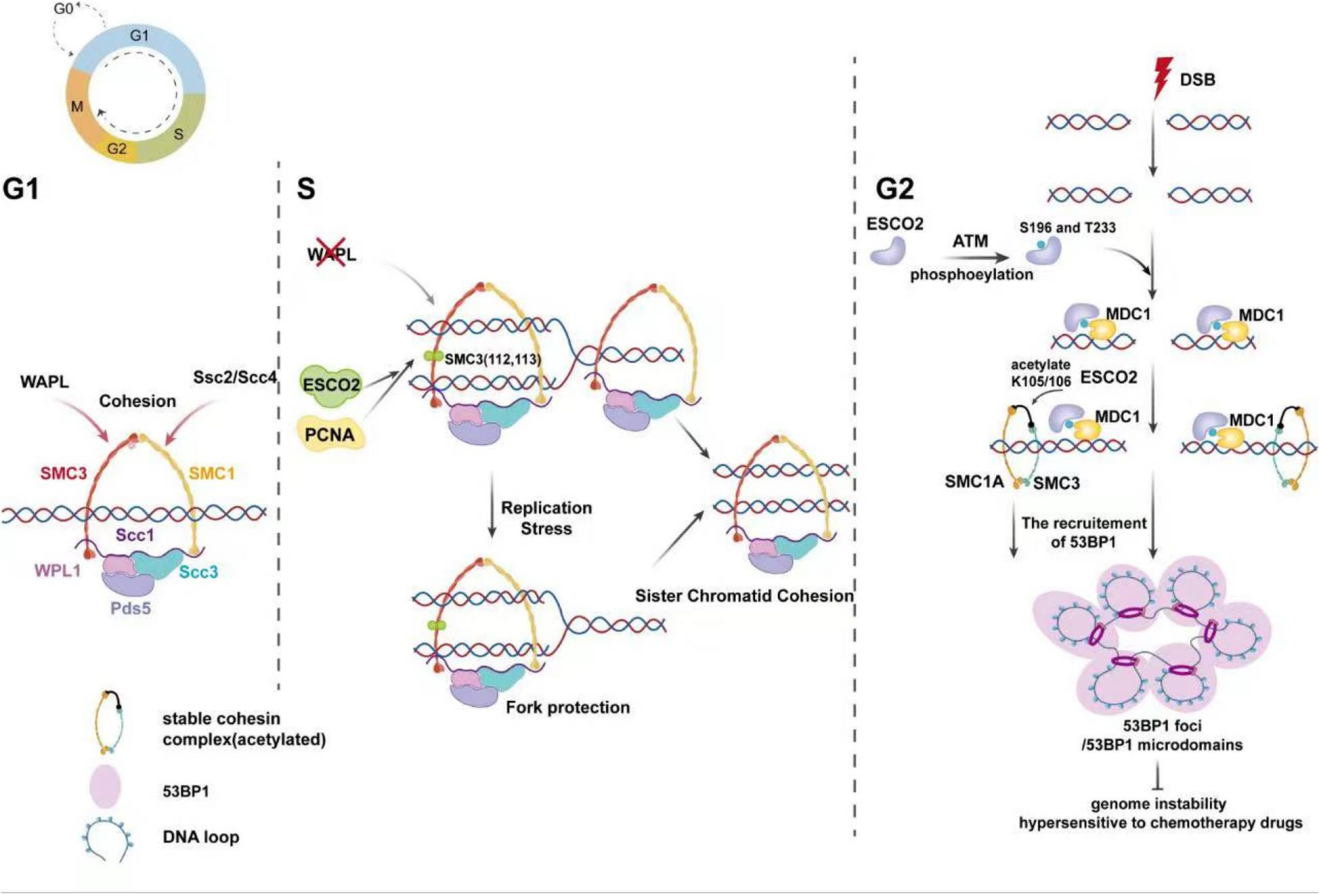
ESCO2 regulates sister chromatid cohesion, DNA double-strand breaks, and cell cycle progression

In vertebrates, ESCO1 and ESCO2, the orthologs of the yeast ECO1 protein [9–12], exhibit divergence in structure, function, potential as therapeutic targets in cancer, and numerous other aspects (Table 1). ESCO2 interacts with proliferating cell nuclear antigen (PCNA) to facilitate sister chromatid cohesion [16–18]. By acetylating the cohesin complex subunit SMC3, ESCO2 promotes the binding of the cohesion-stabilizing factor Sororin, thus counteracting the WAPL-mediated release of cohesin [18–20]. The cohesin complex, a multi-subunit structure, ensures proper sister chromatid cohesion until late cell-cycle stages, preventing early chromatid separation and guaranteeing accurate chromosome segregation (Fig. 1) [21, 22]. Moreover, the regulation of three-dimensional chromatin organization is significantly influenced by cohesin [23], which also plays an essential role in repairing DNA damage, especially through facilitating homologous recombination mechanisms [24]. Among different DNA damage types, DNA double-strand breaks (DSB) pose one of the greatest hazards to the genomic stability of mammalian cells. ESCO2 undergoes phosphorylation by Ataxia Telangiectasia Mutated, subsequently engaging in interactions with mediator of DNA damage checkpoint protein 1 (MDC1). MDC1 subsequently localizes ESCO2 to sites of DNA double-strand breaks, where ESCO2 acetylates lysine residues K105 and K106 of SMC3, thus reinforcing cohesin stability. This promotes the formation of 53BP1 foci (regions of 53BP1 protein aggregation), contributing to DNA damage responses (DDR) and maintaining genome stability (Fig. 1) [25]. This process ensures that cells maintain normal proliferation, differentiation, and other physiological functions.

**Table 1:** Comparative Analysis of ESCO1 and ESCO2

Items	ESCO1	ESCO2
Domain [13]	N-terminus: contains a classic proliferating cell nuclear antigen (PCNA)-interacting motif (PIP box) and directly binds to PCNA through its PIP box to achieve replication-coupled acetylation of SMC3 c-terminus: contains a highly conserved acetyltransferase domain	n-terminus: contains one or more zinc finger (ZnF) domains c-terminus: contains a highly conserved acetyltransferase domain.
Cell cycle [13]	Esco1 is present at nearly constant levels throughout the cell cycle	Esco2 levels are low in G1, and only increase as APC activity drops during S phase
Distinct chromosomal addresses	Colocalization of Esco1 with the insulator protein CTCF and cohesin at the base of chromosome loops. [14]	It is near the active DNA replication forks and is recruited to the chromatin through the CRL4^MMS22L^ complex (regulated by GSK3 phosphorylation) [15]
Function [10]	① Esco1 is responsible for most Smc3 acetylation but has very little effect on sister cohesion ②It does not support sister chromatid cohesion	① It enables the acetylation of the SMC3 subunit within the cohesin complex, facilitating the establishment of sister chromatid cohesion ②ESCO2 supports sister chromatid cohesion.
Expression in tumors	Currently, definitive evidence establishing ESCO2's​​ disease-specific aberrant expression ​​or​​ its direct role in driving malignant phenotypes in tumors ​​is lacking.​	​​It is expressed​​ in a variety of human malignancies, including​​ gastric cancer, colorectal cancer and so on. ​​Furthermore, its expression level frequently exhibits significant associations with​​ tumor stage, grade, invasiveness, metastatic potential, and poor prognosis
Cancer treatment potential	Currently, it has rarely been studied as a target for tumor treatment	Promising to become a new tumor treatment target

Recently, an increasing number of studies have demonstrated the importance of ESCO2 in tumorigenesis [26–29]. ESCO2 has been identified as a critical factor influencing the progression of various human cancersESCO2 [26, 30]. Clinical studies have shown significant upregulation of ESCO2 in diverse human tumors, including gastric cancer (GC) [28], colorectal cancer (CRC) [30, 31], lung adenocarcinoma (LUAD) [27], lung squamous cell carcinoma (LUSC) [32], hypopharyngeal carcinoma (HPC) [33], renal cell carcinoma (RCC) [26, 34], Low Grade Glioma (LGG) [35], breast cancer [36, 37], melanoma [38]. Clinicopathological studies indicated that ESCO2 expression correlates with tumorigenesis, progression, and patient prognosis.

## Biological functions and characteristics of ESCO2

ESCO2 is a highly conserved endopoly-acetyltransferase, initially identified in budding yeast [39]. Its activity and expression levels are strictly regulated by the cell cycle. At different cell cycle stages, the phosphorylation state and protein stability of ESCO2 change, affecting its interaction with other cell cycle regulatory proteins and its functions. During the S-phase, the increased expression and enhanced activity of ESCO2 promote sister-chromatid cohesion, ensuring proper chromatid adhesion and condensation. This function is critical for chromosome stability and accurate genetic material transmission. In the post-mitotic stage, ESCO2 activity is suppressed, facilitating correct chromosome separation during mitosis [40]. Emerging evidence highlights ESCO2’s indispensable role in DDR pathways, particularly double-strand break repair and nucleotide excision repair mechanisms [25]. Structurally, ESCO2 features an N-terminal C2H2 zinc-finger domain and a conserved acetyltransferase domain [39]. The human ESCO2 gene is mapped to chromosome 1q25.2–q25.3, spanning 8.3 kb and consisting of 10 exons interspersed by 9 introns. The encoded protein consists of 604 amino acids (AA), containing a signal peptide composed of 17 AA residues. When ESCO2 function is abnormal, cell-cycle checkpoints activate, causing cells to arrest at specific stages, such as the S-phase, thereby inhibiting cell proliferation. Additionally, abnormalities in ESCO2 result in sister-chromatid cohesion defects, leading to improper chromosome segregation and the generation of aneuploid cells with abnormal chromosome numbers, consequently increasing tumor and disease risk.

## ESCO2 and tumors

### GC

**Fig. 2:**
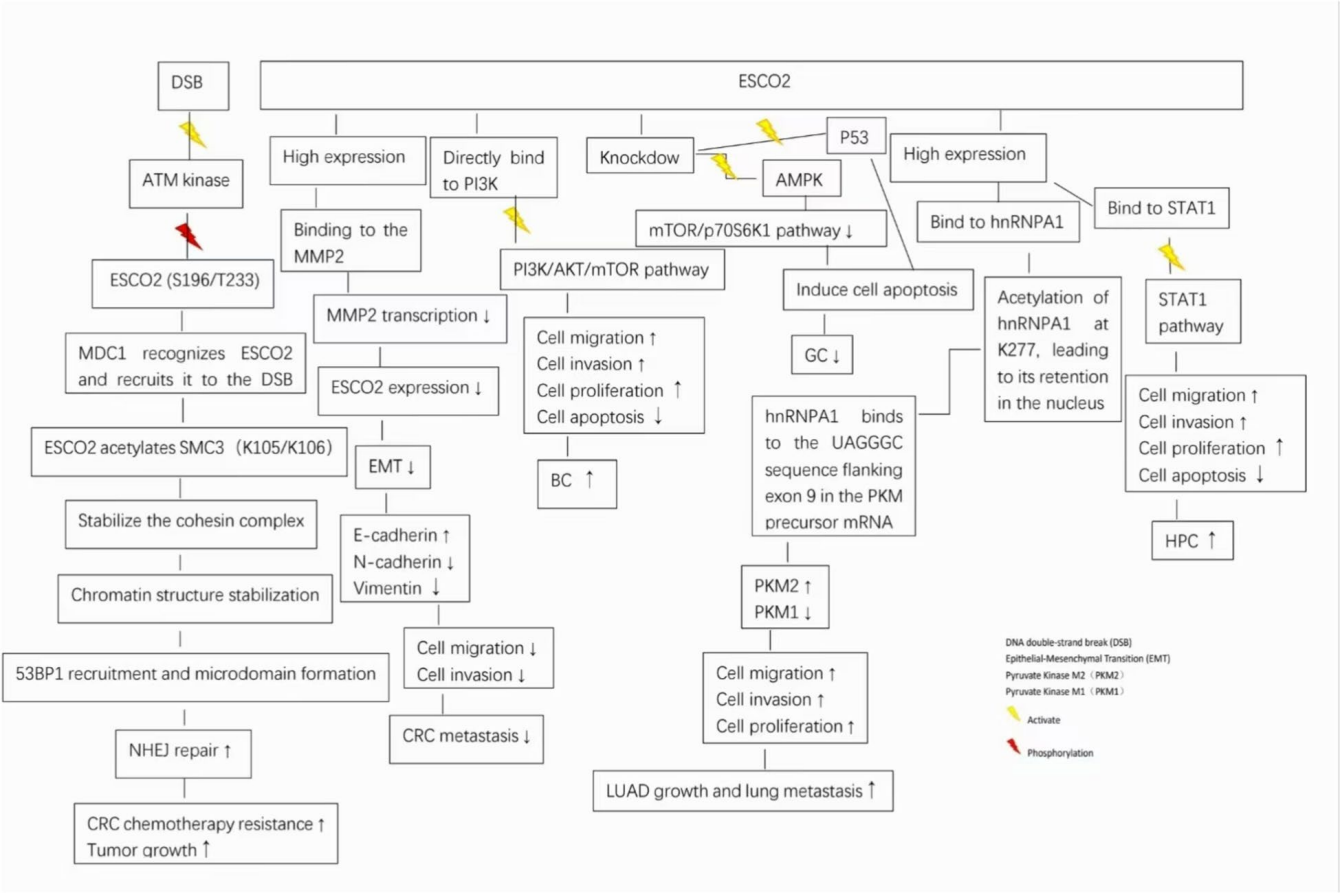
ESCO2 promotes tumor progression through multiple signaling pathways

GC, characterized by high malignancy and aggressiveness, ranks as the fifth most prevalent cancer globally and is responsible for the fourth highest number of cancer-associated deaths worldwide [40–42]. Patients diagnosed with GC face an extremely poor prognosis, The 5 year relative survival rates for different stages of gastric cancer vary significantly: for localized gastric cancer, the survival rate is approximately 75%; for regional gastric cancer, the rate drops to about 35%; and for metastatic gastric cancer, the survival rate is as low as 7% [43]. Chen et al. [28] conducted an analysis comparing ESCO2 expression at the mRNA and protein levels across various human GC cell lines against the GES-1 cell line, a human immortalized gastric epithelial model. Their results indicated that ESCO2 transcription was markedly increased in GC cells, while protein expression exhibited notable variability among cell lines. Additionally, a xenograft tumor model was created through the introduction of BGC-823 cells, previously transfected with a pGCSIL-GFP plasmid carrying siRNA-1, into nude mice. The results demonstrated that downregulation of ESCO2 effectively suppressed tumorigenic potential. Functional assays confirmed that knocking down ESCO2 markedly impaired cell proliferation, inhibited colony formation, and promoted apoptosis in AGS cells in vitro, as well as significantly restrained tumor growth in vivo​​ (Table 2). Mechanistically, In gastric cancer, ESCO2 promotes cell proliferation by modulating the p53 and mammalian target of rapamycin (mTOR) signaling pathways [28]. ESCO2 silencing was found to suppress the mTOR/RPS6K1 signaling cascade while concurrently enhancing the phosphorylation of AMPKα and p53, ultimately leading to decreased proliferation and increased apoptotic activity (Fig. 2). These data indicate a critical role of ESCO2 in gastric carcinogenesis and highlight its potential as a therapeutic target. Nevertheless, studies have revealed significant heterogeneity in ESCO2 protein expression levels across different gastric cancer cell lines. ​​However, the association of this expression heterogeneity with gastric cancer subtypes, differentiation status, or clinicopathological features remains unexplored.​​ Importantly, how to mitigate the impact of this heterogeneity and standardize experimental models (such as through uniform cell line selection or stratified patient sampling) presents critical challenges for mechanistic investigation and clinical translation. ​​While​​ ESCO2 knockdown suppresses the mTOR/p70S6K1 pathway ​​via​​ AMPK activation, ​​it remains unclear whether​​ this activation relies on other signaling molecules. ​​Furthermore​​, ESCO2 knockdown activates the p53 pathway and induces apoptosis, ​​yet​​ key mechanistic details including the ​​specific mode of their interaction​​ (direct binding ​​versus​​ mediation via intermediate molecules) and the ​​precise regulatory mechanisms​​ by which this interaction modulates p53 activity require substantial collaborative investigation.​

**Table 2:** Effects of *ESCO2* expression regulation on cancer

Item	GC [28]			CRC [30]		LUAD [27]		HPC [33]			RCC [26]	
ESCO2 expression	There are differences			↑		↑		↑			↑	
ESCO2 expression												
	High	Knockdown	Silencing	High	Knockdown	High	Knockdown	High	Knockdown	Silencing	High	Knockdown
Tumorigenesis						↑	↓					
Malignant progression				↓		↑	↓			↓		
Metastasis				↓	↑or↓	↑	↓	↑	↓	↓	↑	↓
Cell viability								↑	↓	↓		
Cell proliferation	↑	↓			↓	↑	↓	↑	↓			
Invasion				↓	↓	↑	↓				↑	↓
Cell apoptosis	↓	↑	↑		↑			↓	↑			
Cell growth			↓			↑	↓			↓	↑	↓
Overall survival				↑		Negative correlation					↓	
Death rates						↑	↓					
Disease-free survival				↑		Negative correlation						

“↑” Denotes *ESCO2* upregulation in specified tumors (gastric cancer, colorectal cancer, lung adenocarcinoma, hypopharyngeal cancer, renal cancer), ​​associated with​​ promoted proliferation, invasion, metastasis, and tumorigenesis/progression, along with relatively prolonged​​ overall survival

“↓” Indicates *ESCO2* downregulation, linked to suppressed proliferation, invasion, metastasis, and tumorigenesis/progression, ​​and relatively shortened overall survival​​

### CRC

CRC represents nearly 10% of cancer cases globally, contributing to roughly 9% of all cancer-related mortalities worldwide [44]. Among individuals younger than 50 years, CRC is now the primary cause of cancer-related death among males and ranks second in females, surpassed only by breast cancer [45]. Both CRC incidence and related mortality rates have exhibited a continuous upward trend recently [44, 45]. By examining data available in the Oncomine database, Guo et al. [30] observed increased ESCO2 expression levels in CRC samples. To further elucidate the clinical implications of ESCO2 expression in CRC, 376 patients were categorized into two groups (high or low expression) based on the median ESCO2 level. Analyses of ESCO2 expression were conducted to investigate correlations with clinicopathological features. Additionally, ESCO2 mRNA expression levels were compared between CRC patient groups stratified by lymph node status and tumor stage. The results demonstrated that low ESCO2 expression in CRC patients was frequently associated with advanced tumor stage, increased invasion, and distant metastatic events. Specifically, data from the Hexian Memorial Hospital (HMH) cohort, comprising 211 fresh CRC tissues and 106 paraffin-embedded CRC samples collected between January 2003 and December 2008, indicated that low ESCO2 expression correlated significantly with poorly differentiated tumors (*P = *0.021), increased tumor invasion depth (*P* = 0.028), lymphovascular invasion (*P = *0.011), and distant metastasis (*P* = 0.035). Furthermore, reduced ESCO2 levels were linked to decreased overall survival (OS) and progression-free survival (PFS) (Table 2). Functionally, silencing ESCO2 enhanced the invasive properties of CRC cells, whereas its ectopic overexpression diminished invasive potential. Mechanistically, ESCO2 directly binds to the MMP2 promoter, inhibits its activity, significantly downregulates MMP2 expression, blocks epithelial-mesenchymal transition (EMT), and suppresses cancer metastasis [30, 46]. Furthermore, circular RNA methyltransferase-like 15 (circMETTL15) acts as a tumor-promoting factor by binding miR-374a-5p, enhancing ESCO2 expression and thus promoting CRC progression. Luo et al. [31] reported that circMETTL15 expression was significantly upregulated in CRC tissues and cell lines, correlating closely with larger tumor dimensions, advanced TNM staging, and increased lymphatic spread. Their experimental findings showed that silencing circMETTL15 suppressed LoVo cell proliferation, invasion, and epithelial-mesenchymal transition (EMT), while simultaneously inducing apoptosis. Thus, ESCO2 holds promise as a novel therapeutic target in CRC management. Guo et al. [30] reported upregulated ESCO2 expression in CRC tissues correlated with metastasis and poor prognosis, positioning it as an oncogenic driver. Conversely, Luo et al. [31] demonstrated that ESCO2 knockdown suppressed CRC cell proliferation, invasion, migration, and EMT, while promoting apoptosis–suggesting tumor-suppressive properties (Fig. 2). This apparent contradiction in expression-phenotype relationships remains unresolved and necessitates investigation into the following mechanisms: Does ESCO2 exert bidirectional effects during distinct stages of tumor progression? Could phosphorylation, acetylation, or other PTMs dynamically modulate ESCO2’s functional states? These represent fundamental bottlenecks in understanding ESCO2’s role in CRC biology and its potential as a therapeutic target.

### LUAD

Lung cancer (LC) is currently the most commonly diagnosed malignant tumor and is the most common cause of death caused by cancer in the world [47]. Lung cancer has the leading cancer incidence in the world [48]. Non-small cell lung cancer (NSCLC) constitutes approximately 85% of all LC cases. LUSC, a common histological subtype of NSCLC, accounts for about 30% of cases [49]. LUAD, representing 35–40% of LC cases, is characterized by high morbidity and low survival rates [50]. Zhu et al. [27] investigated the expression pattern of ESCO2 in LUAD through multiple public databases, including Oncomine, GEO, and TCGA, and confirmed its elevated expression in LUAD tissues. Further experiments, including proliferation assays, colony formation tests, migration and invasion analyses in vitro, as well as xenograft studies in vivo, indicated that ESCO2 overexpression could significantly enhance malignant behaviors of LUAD cells, whereas ESCO2 knockdown markedly suppressed tumor growth and metastasis (Table 2). Regarding underlying mechanisms, ESCO2 physically associates with the RNA-binding protein hnRNPA1 and mediates its acetylation at lysine residue K277, thereby facilitating nuclear retention of hnRNPA1. Nuclear hnRNPA1 influences the alternative splicing of pyruvate kinase (PKM) [28, 51], shifting the balance toward the glycolysis-promoting isoform PKM2 rather than PKM1 [52]. Consequently, the increased PKM2 level accelerates aerobic glycolysis, promoting metastatic potential and cellular migration of LUAD cells (Fig. 2). Zhang et al. [32] conducted extensive bioinformatic analyses, identifying eight candidate genes potentially implicated in the pathogenesis of LUSC. Their findings indicated these genes significantly correlate with methylation at TRIM58/cg26157385 and treatment outcomes, highlighting their potential as diagnostic markers. Thus, ESCO2 may represent a promising diagnostic and therapeutic target for NSCLC. However, current research on LUSC primarily relies on bioinformatics analyses without sufficient in vitro or in vivo validation. Identifying the upstream regulatory factors (such as transcription factors, epigenetic modifications, or signaling pathways) that drive its overexpression through in vitro or in vivo experiments is an immediate and long-term research goal for clinical researchers.

### HPC

HPC, originating from epithelial cells within hypopharyngeal mucosal tissues, constitutes approximately 5% of all Head and neck cancers, with an overall five-year survival rate below 20% [53]. Given that HPC frequently presents at advanced clinical stages, the prognosis of patients remains particularly unfavorable [54]. Through an analysis utilizing the TCGA dataset, Hu et al. [33] found that ESCO2 was markedly upregulated in HPC tissues relative to normal controls. Patients were grouped according to ESCO2 expression levels (high or low) to investigate associations with tumor progression. Results indicated that high ESCO2 expression was associated with increased distant metastasis and lymph node metastasis (*P* < 0.05) [27, 33]. Further functional studies involving proliferation, migration, apoptosis assays in vitro, as well as xenograft tumor models in nude mice, demonstrated that ESCO2 depletion significantly attenuated tumor growth and metastatic capacity (Table 2). At a mechanistic level, downregulation of ESCO2 induced the upregulation of STAT1 expression. Compared with controls, proliferation was inhibited following ESCO2 silencing. However, STAT1 overexpression significantly reversed the inhibition of proliferation induced by ESCO2 depletion. Cell viability assays also indicated that the reduction in viability following ESCO2 silencing was attenuated by STAT1 overexpression. Consistently, silencing ESCO2 inhibited cellular migration; however, ectopic overexpression of STAT1 effectively rescued this impairment, indicating that STAT1 is a critical mediator downstream of ESCO2 in HPC development (Fig. 2) [9, 33]. Taken together, these data suggest that ESCO2 represents a potential therapeutic target for treating HPC. However, the mechanistic details of how ESCO2 regulates STAT1 remain elusive. Additionally, the FaDu cell line alone cannot fully represent the heterogeneity of HPC. Future studies should incorporate diverse models and focus on elucidating the ESCO2-STAT1 interaction mechanism and expanding downstream signaling pathways. Since STAT1 overexpression can restore cell migration, the potential for therapeutic synergy through targeting dual pathways represents an intriguing direction for further investigation.

### RCC

RCC is ranked second globally among genitourinary malignancies, showing a continuous rise in morbidity and mortality rates, which increased by 2.2% and 1.8% respectively in 2018 [55]. As one of the most prevalent renal malignancies, RCC has garnered considerable attention. Wang et al. [26] conducted a comparative analysis of ESCO2 expression between 289 RCC samples and 32 normal controls. The results revealed significantly elevated ESCO2 expression in RCC, and higher ESCO2 expression correlated with poorer patient prognosis (*P* < 0.01). ESCO2 expression correlated significantly with age, sex, pathological stage, primary tumor status, and regional lymph node involvement (all *P <  *0.05). In vitro studies revealed that ESCO2 knockdown significantly inhibited RCC cell proliferation, invasion, and migration [56] (Table 2). Mechanistically, Western blot analyses showed that expression of phosphorylated AKT/mTOR pathway proteins decreased after ESCO2 silencing, whereas total AKT and mTOR levels remained unchanged [57]. Thus, the AKT/mTOR pathway may represent a key pathway regulated by ESCO2 in RCC. Additionally, ESCO2 silencing reduced expression of PCNA and p53. As a major tumor suppressor, p53 interacts with the AKT/mTOR pathway [9, 58]. Percival et al. [37] demonstrated that simultaneous knockdown of p53 and ESCO2 induced abnormal chromosome segregation, genomic instability, and defective sister-chromatid cohesion. Following si-ESCO2 in RCC cells, p53 expression was downregulated, suggesting potential interactions between ESCO2 and p53 during RCC development. Zhang et al. [34] explored data from TCGA comprising RCC samples and 72 normal tissues, discovering a marked upregulation of long non-coding RNA ZFPM2-AS1 in RCC [34, 59]. Subsequently, 60 RCC tumor specimens were stratified into two groups, high and low, based on median ZFPM2-AS1 expression. Elevated ZFPM2-AS1 expression significantly associated with advanced TNM stage, and worse prognosis, but exhibited no significant relationship with age or gender. Functional assays indicated that silencing ZFPM2-AS1 impeded cell proliferation and migration, while promoting apoptosis. Additionally, expression of miR-130a-3p, negatively correlated with ZFPM2-AS1, was reduced in RCC samples, suggesting that ZFPM2-AS1 downregulated miR-130a-3p. Furthermore, miR-130a-3p directly targeted ESCO2 mRNA, negatively regulating its expression. Overexpressing miR-130a-3p significantly reduced ESCO2 levels at both mRNA and protein scales, confirming ESCO2 as a downstream regulatory target. Moreover, suppression of miR-130a-3p or overexpression of ESCO2 reversed the tumor-suppressive effects triggered by ZFPM2-AS1 silencing. These findings indicate ZFPM2-AS1 facilitates RCC progression through modulation of the miR-130a-3p/ESCO2 pathway [34]. Although studies have demonstrated that ESCO2 knockdown reduces expression of p-AKT, p-mTOR, PCNA, and p53, thereby suppressing malignant behaviors in renal cancer cells, ​​the mechanisms underlying their coordinated regulation by ESCO2 require further investigation.​​ Given that ZFPM2-AS1 upregulates ESCO2 ​​by sponging​​ miR-130a-3p to promote RCC progression, ​​future research should explore therapeutic interventions targeting this axis.​​ Potential strategies include ​​inhibiting​​ ZFPM2-AS1 expression, ​​enhancing​​ miR-130a-3p activity, or ​​directly targeting​​ ESCO2, ​​offering novel therapeutic approaches​​ for RCC treatment.

### Other tumors

Guo et al. [35] conducted a pan-cancer analysis of ESCO2 using the UCSC Xena database to evaluate its expression across various cancer types. Results demonstrated that ESCO2 was significantly upregulated in most cancers. Prognostic analyses assessed correlations between ESCO2 expression and outcomes. High ESCO2 expression was an important risk factor for poor OS in cancers such as low-grade LGG and adrenocortical carcinoma (ACC). To exclude potential bias arising from deaths unrelated to tumors, Disease-Specific Survival Analysis (DSS) were carried out. Results demonstrated that elevated ESCO2 expression correlated with reduced DSS in various cancers including LGG, ACC, and others. Furthermore, increased ESCO2 levels were linked to alterations in Disease-Free Interval across multiple cancers. Elevated ESCO2 expression also corresponded to poorer Progression-Free Interval outcomes in patients diagnosed with various cancers [35]. Thus, these observations underscore ESCO2’s potential as a prognostic biomarker across multiple tumor types.

Moreover, analyses examining associations between ESCO2 and immune-related markers, notably CD274, indicate that ESCO2 may modulate the effectiveness of immunotherapy. CD274 overexpression is known to suppress anti-tumor immune activity and promote malignant tumor progression, survival, and proliferation [60]. Tumor mutational burden was positively correlated with ESCO2 expression in cancers such as LGG, LUAD and Colon Adenocarcinoma (COAD), whereas it exhibited negative correlations in Thyroid Carcinoma (THCA) and thymoma. Microsatellite instability analyses showed positive associations with ESCO2 in cancers including COAD, soft tissue sarcoma, salivary adenocarcinoma, and gastric adenocarcinoma, and negative correlations in THCA.

Liu et al. [61] conducted integrative analyses combining transcriptomic profiles with corresponding clinical data from 1064 LGG cases sourced from TCGA and CGGA databases. Results indicated that ESCO2 expression correlated with recurrence and tumor grade in LGG. ESCO2 acted as an independent risk factor, significantly reducing patient OS. It may exert oncogenic effects by influencing cellular replication and DNA repair.

## Conclusion

In summary, ESCO2 establishes sister chromatid cohesion, functioning similarly to a “molecular glue” that maintains firm chromatid adhesion. By participating in chromatid cohesion, ESCO2 indirectly influences chromosome segregation. Furthermore,​​ ESCO2 acetylates the SMC3 subunit of the cohesin complex, ​​thereby​​ stabilizing its binding to chromatin, ​​which promotes​​ the repair of DSB and maintains​​ genomic stability. ​​In cancer cells​​, enhanced DSB repair capacity ​​accelerates​​ DNA replication rates ​​and facilitates​​ evasion of apoptosis. ​​Notably​​, studies ​​have observed​​ that ESCO2 ​​exhibits context-dependent functions​​ across various tumor types. ESCO2 is significantly upregulated in kidney cancer tissues, and ESCO2 knockdown inhibits cancer cell growth, invasion, and migration by regulating the AKT/mTOR pathway [26]. Zhu et al. also demonstrated that ESCO2 could promote LUAD cell proliferation and metabolic reprogramming of metastasis in vitro and in vivo [27]. However, ESCO2 has been shown to inhibit cancer metastasis in colorectal cancer by reducing MMP2 expression [30]. Chen et al. determined that ESCO2 was significantly upregulated in HCC tissues and linked to a poorer prognosis. Knockdown of ESCO2 significantly inhibited HCC cell proliferation both in vivo and in vitro. Most notably, ESCO2 could promote the PI3K/AKT/mTOR pathway, accelerating the cell cycle and inhibiting apoptosis, and thus increasing HCC growth [22]. The PI3K/Akt/mTOR signaling pathway is indispensable in many cellular biological processes, including cell proliferation, survival, metabolism, motility, angiogenesis, and response to stress and therapy. Extensive research supports its critical role in regulating tumor growth, metabolism, metastasis, and treatment resistance [62, 63]. Collectively, these findings establish​​ ESCO2 ​​as a pivotal regulator​​ in multiple critical aspects of tumorigenesis, ​​including​​ tumor progression, metastasis, and therapy resistance. ​​This multifaceted involvement underscores its significant potential as a promising therapeutic target.​​ Consequently, ​​the development of effective ESCO2 inhibitors holds promise for​​ precisely targeting the survival and progression pathways of cancer cells. ​​Such advancements could pave the way for novel therapeutic approaches,​​ offering a ​​promising avenue​​ in the ongoing battle against cancer.

## Supplementary Information

Supplementary Material 1

## Data Availability

No datasets were generated or analysed during the current study.
