# Supplementary figures and images for: The expression of establishment of cohesion 1 homolog 2 (ESCO2) in tumor cells and its research progress as a therapeutic target
Source: Eur J Med Res. 2025 Oct 6;30:930. doi: 10.1186/s40001-025-03164-4 (PMC12502477; doi:10.1186/s40001-025-03164-4)

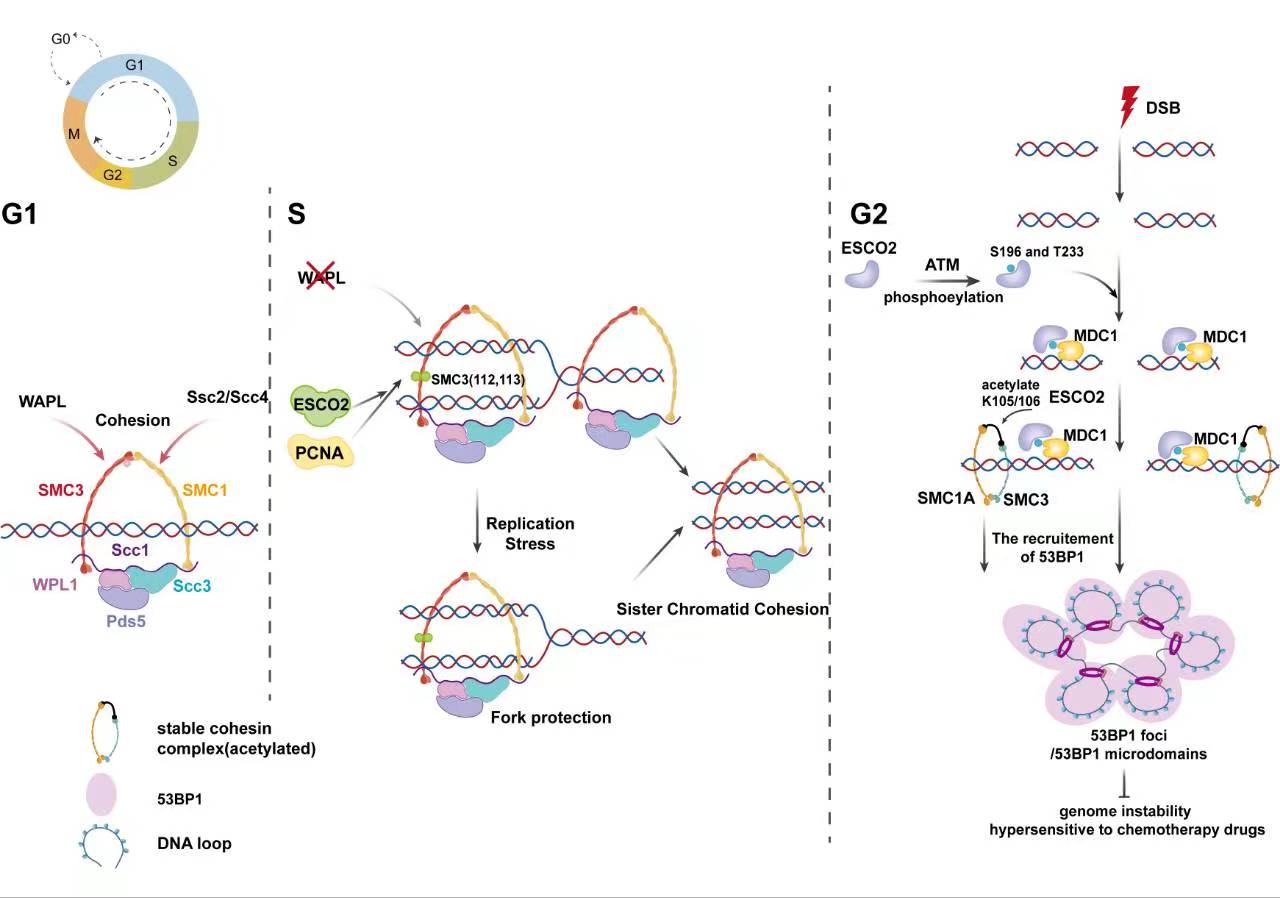


**Figure 1. Figure of ESCO2-related cell cycle events**


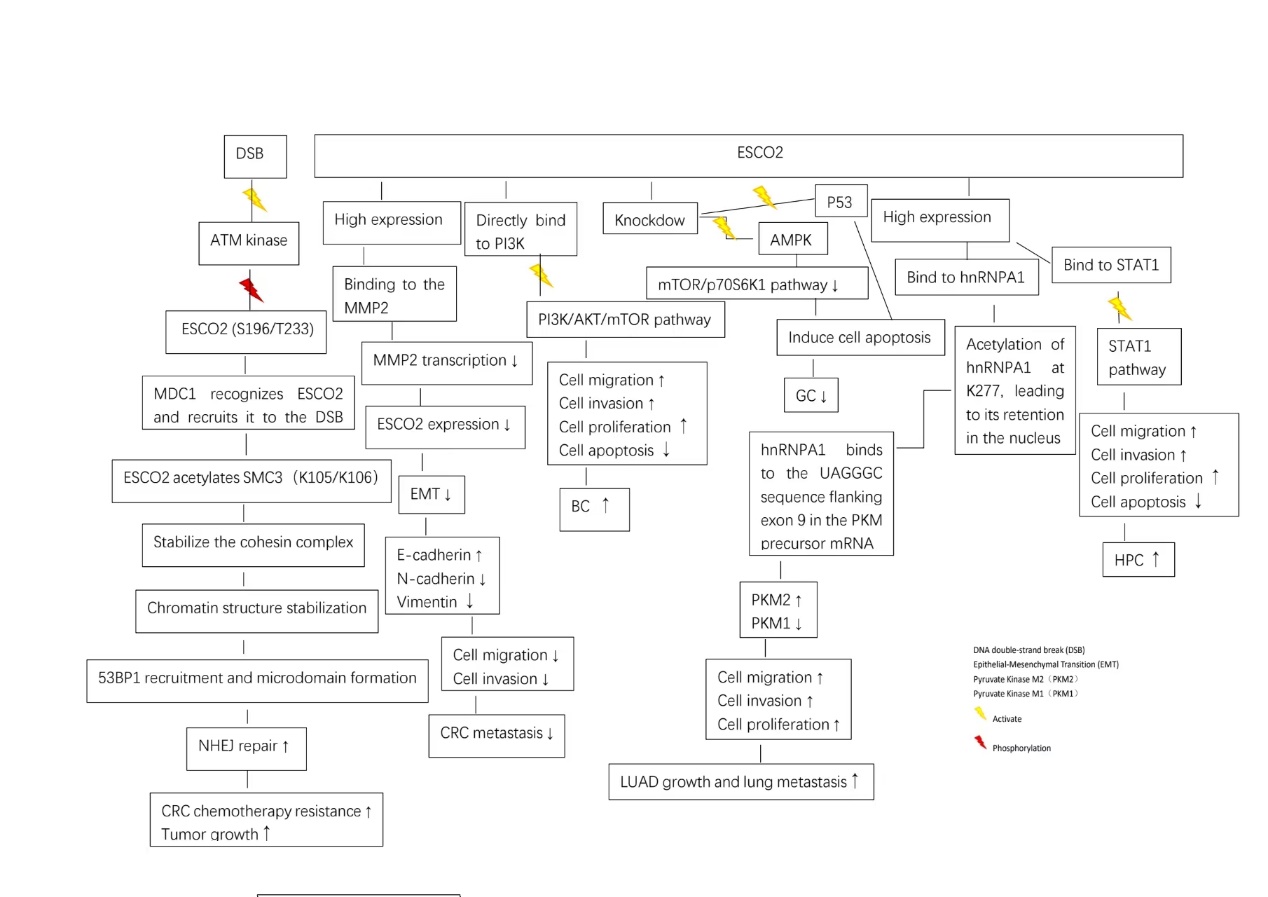


**Figure 2. Figure of ESCO2 interacts with tumors**

Supplement: Supplementary file 1 — Supplementary Material 1 [file 40001_2025_3164_MOESM1_ESM.docx]
